# Haematological Monitoring in Clozapine Use: Blood Best Sampled in the Afternoon?

**DOI:** 10.1192/bjo.2022.77

**Published:** 2022-06-20

**Authors:** Adedoyin Ade-Dosumu, Henry O'Connell

**Affiliations:** 1 Laois/Offaly Mental Health Services, St Fintan's Hospital, Portlaoise, Co. Laois, Ireland.; 2 University of Limerick, Limerick, Ireland

## Abstract

**Aims:**

Clozapine is an atypical antipsychotic used in the management of Treatment-Resistant Schizophrenia (TRS). Despite its efficacy, Clozapine is associated with rare but clinically important haematological side effects of neutropenia and agranulocytosis, thus warranting regular monitoring of full blood count (FBC) white blood count (WBC) and neutrophil count (NC).

Clozaril Patient Monitoring service (CPMS) supervises the prescribing of Clozapine and haematological testing for patients. Amber and Red alerts are issued by the CPMS when WBC and NC values fall below specified levels, Clozapine treatment is either suspended or discontinued completely.

**Methods:**

We present a case of 35-year-old Caucasian lady with a history of schizophrenia who was maintained on Clozapine treatment for 10 years and whose Clozapine was necessarily stopped because of red alerts. This lead to a significant deterioration in her mental state and level of functioning necessitating a prolonged hospital admission.

**Results:**

- Detailed retrospective review of this patient's CPMS results over a 2-year period identified consistently higher levels of WBC and NC, when the blood tests were conducted in the afternoon compared to mornings.
- Literature review supported the phenomenon of a diurnal variation of WBC and NC levels in a proportion of patients prescribed Clozapine.
- With clear knowledge of this patient's diurnal trends in WBC and NC count, it was possible to liaise with CPMS and restart her Clozapine treatment, leading to a significant improvement in her mental state and level of functioning.

**Conclusion:**

- We recommend that clinicians consider this phenomenon of diurnal variation in blood parameters in patients with frequent amber and red alert results who are at risk of having their Clozapine medication discontinued.
- Close collaboration with CPMS and haematological advice may lead to a more nuanced approach to blood sampling, whereby afternoon samples are used in certain at-risk patients.

